# A novel fluorescent probe for detecting hydrogen sulfide in osteoblasts during lipopolysaccharide-mediated inflammation under periodontitis

**DOI:** 10.1038/s41598-021-99761-4

**Published:** 2021-10-11

**Authors:** Xiaoya Lu, Hanchuang Zhu, Yi Chen, Yue Wu, Dongsheng Zhang, Baocun Zhu, Shengyun Huang

**Affiliations:** 1 Department of Oral and Maxillofacial Surgery, Shandong Provincial Hospital, Cheeloo College of Medicine, Shandong University, Jinan, Shandong 250021 China; 2 School of Water Conservancy and Environment, University of Jinan, Jinan, Shandong 250022 China

**Keywords:** Biotechnology, Biomarkers, Diseases

## Abstract

Periodontitis, one of the most common chronic inflammatory diseases, affects the quality of life. Osteogenesis plays an important role in the disease. There is a connection between hydrogen sulfide (H_2_S) and periodontitis, but according to the study has been published, the precise role of H_2_S in inflammation remains in doubt. The main reason for the lack of research is that H_2_S is an endogenous gasotransmitter, difficult to discern through testing. So, we synthesized a novel fluorescence probe which can detect H_2_S in vitro. By using the novel H_2_S fluorescence probe, we found that H_2_S changes in osteoblasts mainly by cystathionine-γ-lyase, and H_2_S increases under LPS stimulation. H_2_S could be a potential marker for diagnosis of inflammatory diseases of bone, and might help deepen studies of the changes of H_2_S level and promote the progression on the researches about pathogenesis of periodontitis.

## Introduction

Periodontitis as one of the most common chronic inflammatory diseases, afflicting man. It can lead to cause of bone resorption, even worse tooth loss. Under normal physiologic conditions, the balance of osteoclasts and osteoblasts is tightly related to avoid the loss of bone. The breakdown of the balance will cause diseases. Avoiding alveolar bone destruction is an important problem to control the periodontitis. However, the detailed mechanism of periodontitis is still largely unknown.

Lipopolysaccharide (LPS), a major toxic factor of gram-negative bacteria, plays a main role in periodontitis. It can cause periodontitis by modulating the activity of the host defenses^1^, inducing a hypoxic phase^2^ etc., and it eventually stimulates bone resorption^3^. LPS may lead to inflammatory response in osteoblasts and osteoblasts, which may results in a disorder in the balance of osteoclasts and osteoblasts even cell death, leading to accelerating bone loss^4^. For experimental researches, LPS was used to stimulate the rat gingival sulcus every day in order to obtain an experimental periodontitis model by immunizing it with the antigen^5^. LPS treated cells are in a similar situation as well. Halitosis is one of the clinical features of periodontitis, and Hydrogen sulfide (H_2_S) is the main unbearable stinky smell of periodontitis and may play a significant role in its development.

Biothiols are indispensable in human physiology, which are in a vital branch of reactive sulfur species (RSS) family. H_2_S is an endogenous gasotransmitter, which is well-known for its stinky smell like rotten eggs. H_2_S is produced by the sulfur-containing materials cysteine, homocysteine or 3-mercaptopyruvate. H_2_S is transformed by cystathionine-β-synthase (CBS), cystathionine-γ-lyase (CSE) and 3-mercaptopyruvate sulfurtransferase (3-MST)^6^. Most researchers previously believe that H_2_S can promote the pathogenesis of periodontitis, and are hugely harmful to their periodontal tissue^7^. But recently, there is evidence shows that H_2_S might be useful in cell protection. For exogenous H_2_S, it can promote LPS-induced apoptosis of osteoblast cells, which might represent a new direction in the treatment of osteomyelitis^8^. When oxidative damage occurs, H_2_S can increase cell viability and reduce cell apoptosis. H_2_S might have an advantageous effect, because according to the research, NaHS treatment can produce anti-inflammatory effects via NO and TNF-α^9^. Besides, H_2_S can protect cell injury by regulating oxidative stress, mitochondrial function, and inflammation. It also has the ability to potentially prevent bone loss in periodontitis^10^. So, there is a connection between H_2_S and periodontitis, but until now, the precise role of H_2_S in inflammation remains unknown.

**Figure 1:**
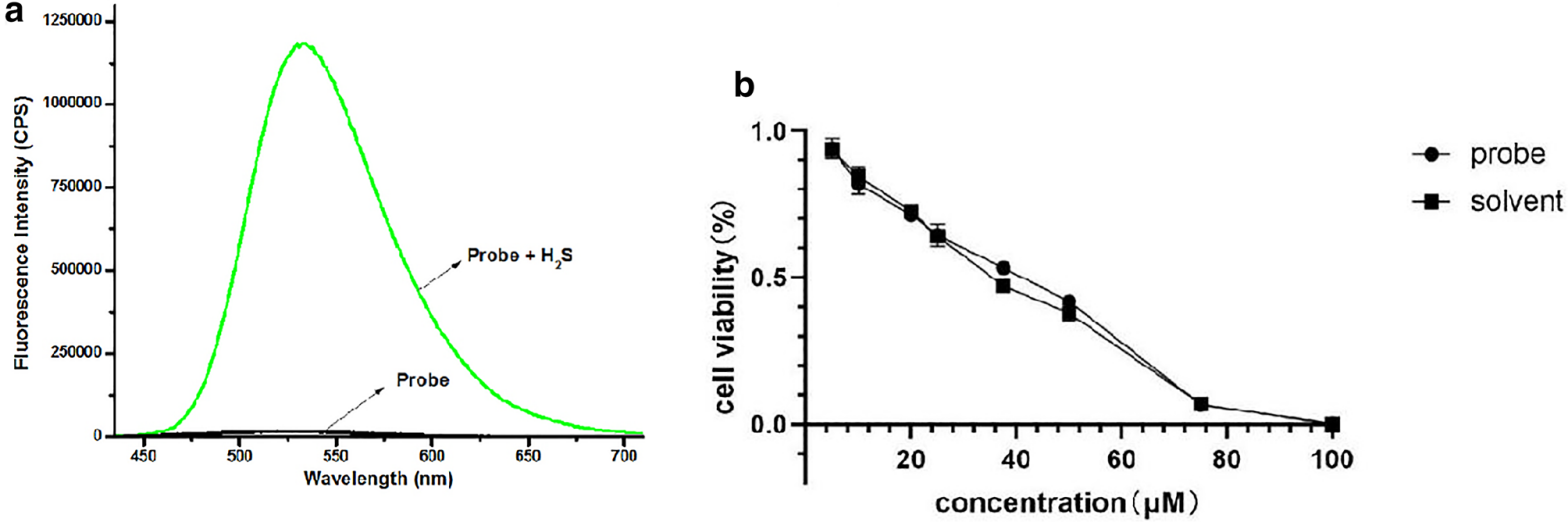
(**a**) The spectral changes of the probe before and after the reaction with H_2_S. (**b**) Toxicity analysis.

Most of the studies focus on the effect of the H_2_S, not many about H_2_S changes under stimulation. Researchers often use Western blot, immunohistochemical staining, and some other methods to detect the H_2_S changes indirectly. Recently, there are some direct techniques to detect H_2_S, such as chromatography, electrochemistry and colorimetry^11^. But a technique that can detect H_2_S directly in living cells is still needed. H_2_S-fluorescence probe, which is high-speed developing, is considered as one of the most helpful instrument areas in the field of H_2_S biology^12^. In recent years, many excellent fluorescent probes have been designed and synthesized through-in-depth-analysis of the structural features of biothiols^13,14^. We previously developed a H_2_S probe, which consists of a 1,8-naphthalimide as fluorophore and azido moiety as recognition site. The introduction of the electron-withdrawing azido group changes the push–pull system and quenches the fluorescence. It is noteworthy that the reaction is easy to carry out and the yield is high. When the probe reacts with hydrogen sulfide, the azido moiety is reduced to an amino group. Because the amino group acts as an electron-donating group, the effect of intramolecular charge transfer is enhanced, and the fluorescence is recovered (Fig. 1a). The probe is able to directly measure the real time H_2_S level in living cells. Overall, because of high resolution and sensitivity of the H_2_S probe make it a helpful tool. There are some studies showing that H_2_S fluorescence probe can detect endogenous H_2_S in real-time and in situ. However, most of them use tumor cells instead of somatic cells, if the probe could be used in somatic cells, it can broaden diagnose and treatment applications of H_2_S. By using a novel H_2_S fluorescence probe, we found that H_2_S changes in osteoblast mainly by CSE, and H_2_S increases under LPS stimulation.

## Materials and methods

### Regents

The hydrogen sulfide fluorescent probe was provided by Professor Baocun Zhu (School of Water Conservancy and Environment, University of Jinan, Jinan, China). 1 mg probe was dissolved in 100 μL dichloromethane, then was diluted with DMSO (Sigma-aldrich, USA) to a final concentration of 1 mM. α-MEM was used to dilute the mother liquor to get different concentrations. The test concentration was 10 μM and the experiment was carried out at room temperature (25 ℃).

DL-propargylglycine (PAG) (cystathionine γ-lyase inhibitor, Sigma-Aldrich), Cysteine (Cys), NaHS, lipopolysaccharide (LPS) (Sigma-aldrich, USA), cell counting kit-8 (CCK-8; Dojindo Molecular Technologies, Tokyo, Japan).

### MC3T3-E1 cell culture

The murine calvaria-derived MC3T3-E1 osteoblast-like cell line (Procell CL-0378, subclone 14) was provided by Procell Life Science and Technology CO., Ltd. Cells were seeded at 5 × 10^4^ cells/mL into 25 cm_2_ flasks and maintained in α-MEM, supplemented with 10% fetal bovine serum (FBS) and 1% penicillin/streptomycin. Cells were maintained in an incubator containing a 5% carbon dioxide/air environment at 37℃.

### Toxicity analysis

The influence of the H_2_S probe on MC3T3-E1 cell was examined by CCK-8. Briefly, MC3T3-E1 cells, seeded at a density of 5 × 10^4^ cells·/ml on a 96-well plate, were maintained at 37 ℃ in a 5% CO_2_, 95% air incubator for 24 h. Then the cells were incubated with different concentrations (0, 5, 10, 20, 25, 37.5, 50, 75 and 100 μM) of probe suspended in culture medium for 24 h. Same as the probe group, the other plate of cells was incubated with same concentrations (0, 5, 10, 20, 25, 37.5, 50, 75 and 100 μM) of solvent. Subsequently, CCK-8 solution was added into each well for 2 h. The absorbance at 450 nm was then measured.

### Application of H_2_S probe to access exogenous H_2_S levels

The cells were pre-treated with NaHS (50, 100, 150, 500 μM) for 30 min, then, treated with the H_2_S probe (10 μM) for 30 min. Fluorescence and bright field images were collected after PBS washing for three times. Green fluorescence was observed under the confocal microscope at excitation wavelengths of 405 nm. In order to control exposure, Smart Gain was kept at the same voltage in every photographs.

### Application of H_2_S probe to access endogenous H_2_S levels

In the periodontium of mammalian host, H_2_S is produced using Cys mainly by CSE and CBS. The cells were pre-treated with Cys (100 μM, 200 μM) for 30 min, then, treated with the H_2_S probe (10 μM) for 30 min. Fluorescence and bright field images were collected after PBS washing for three times. Green fluorescence was observed under the confocal microscope at excitation wavelengths of 405 nm. In order to control exposure, Smart Gain was kept at the same voltage in every photographs.

PAG is an irreversible inhibitor of CSE. It can block the produce of endogenous H_2_S in MC3T3-E1. Therefore, we pre-treated cells with 50 μM PAG, 30 min, then cells were treated with or without Cys for 30 min. Last, fluorescence was examined as before, Smart Gain was kept at the same voltage in every photographs.

Addition of lipopolysaccharide (LPS) for inducing inflammation and assessment with H_2_S probe: The cells were incubated with 1, 2 μg/mL LPS for one day. Subsequently, the culture dish was washed with PBS for three times and incubated with 10 μM probe for 30 min. Then, the cells were washed with PBS, then the fluorescence imaging was examined by confocal microscope, Smart Gain was kept at the same voltage in every photographs.

## Results

Probe spectra and toxicity analysis: As shown in Fig. 1a, the probe itself had almost no fluorescence, but it showed a significant fluorescence enhancement after the addition of H_2_S (100 μM). The cell’s viable and healthy during the detection is a key concern. Figure 1b showed that cell viability was almost not affected by the probe at 10 μM. Toxicity is mainly introduced by solvent, DMSO and dichloromethane. The result verify that the H_2_S probe is harmless to the cell. Thus, the H_2_S probe can be used in living cells for fluorescence imaging analysis.

**Figure 2:**
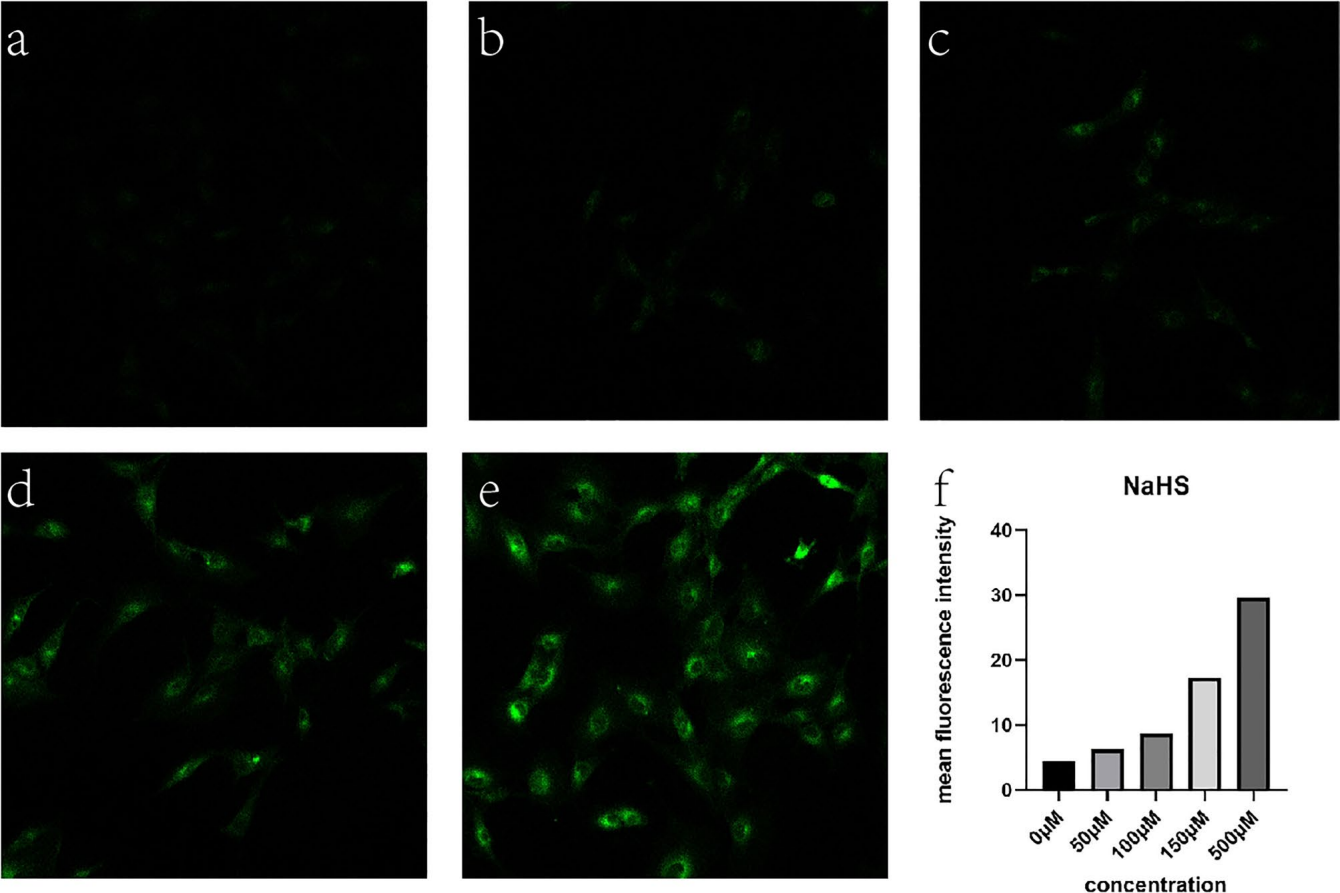
Cell fluorescence imaging of different concentrations of exogenous H_2_S (magnification 10). (**a**–**e**) Fluorescence imaging of cells incubated with different concentration of NaHS (0, 50, 100, 150, 500 μM) and probed by the H_2_S probe (10 μM) for 30 min. (**f**) Fluorescence intensity analysis.

Cell fluorescence imaging of different concentrations exogenous H_2_S: As shown by Fig. 2, with the different concentrations (0, 50, 100, 150, 500 μM) of NaHS, a gradual increase of intensive green fluorescence was observed using 405 nm as an excitation wavelength. Consistent with previous studies, the amount of H_2_S is one third of exogenous of NaHS. Thus, the probe was estimated detection of accuracy to 10 μM. Fluorescent intensity is stable during the progress of taking pictures under the confocal laser scanning microscopy. That indicated that our probe is sensitive to H_2_S, and it also prove that H_2_S probe was cell membrane permeable and can be used in the normal cells for detecting intracellular H_2_S.

**Figure 3:**
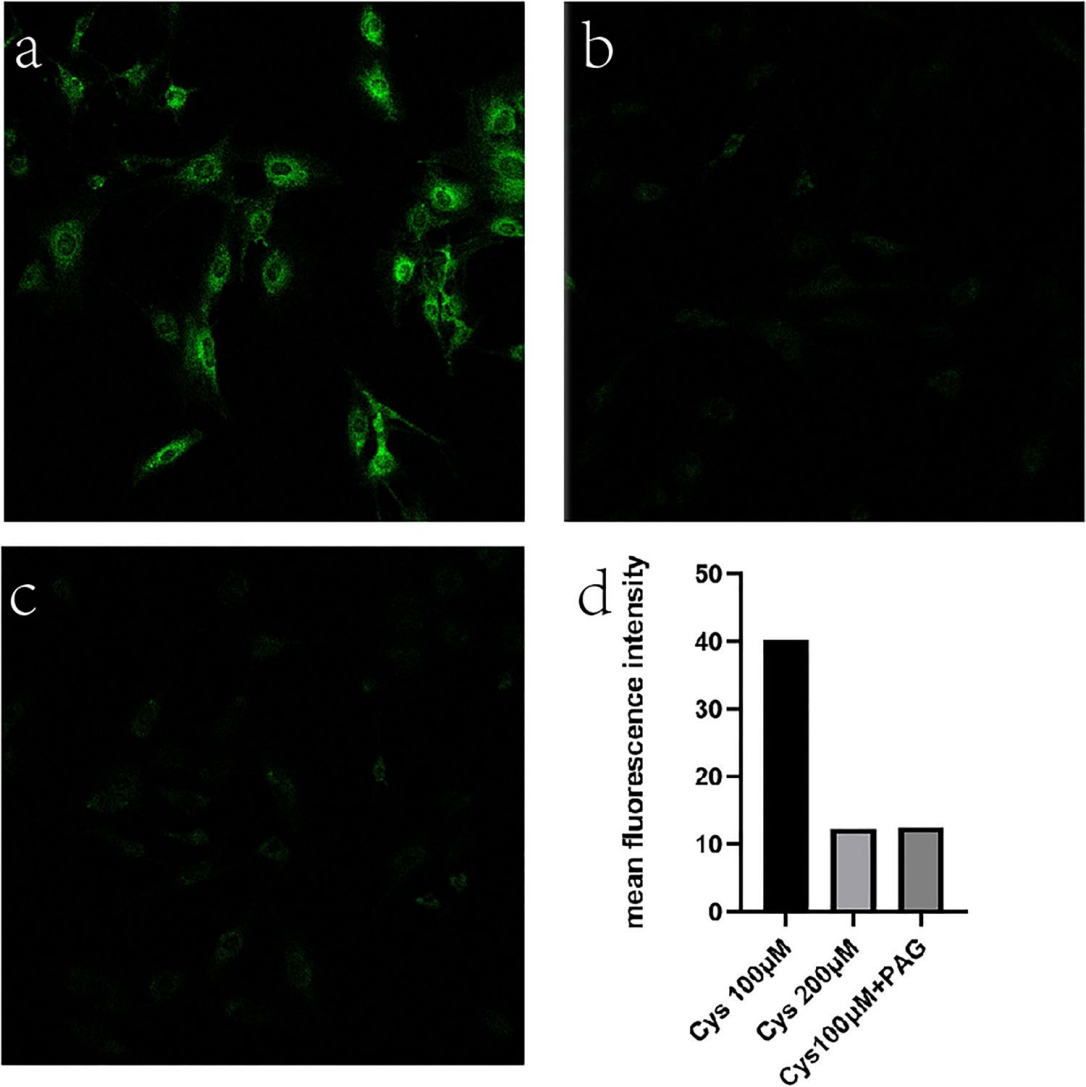
Laser confocal microscope images of H_2_S probe in MC3T3-E1 cells (magnification 10). Cell fluorescence imaging of endogenous H_2_S. (**a**) Fluorescence image of MC3T3-E1 cells incubated with Cys 100 μM for 30 min, then incubated with H_2_S probe (10 μM) for 30 min. (**b**) Fluorescence image of MC3T3-E1 cells incubated with Cys 200 μM for 30 min, then incubated with H_2_S probe (10 μM) for 30 min. (**c**) pre-treated MC3T3-E1 cells with 50 μM PAG for 30 min, then cells were treated with or without Cys for 30 min, then incubated with H_2_S probe (10 μM) for 30 min. (**d**) Fluorescence intensity analysis.

Cell fluorescence imaging of endogenous H_2_S: According to the previous research, for osteoblasts, CSE-H_2_S might be the major path for the H_2_S produced^13^. As shown by Fig. 3, the incubation of cells with 100 μM Cys produced intensive green fluorescence, but the fluorescence decreased when cells were incubated with 200 μM Cys. That means that low dose of Cys could increase H_2_S production, but high dose of Cys inhibited H_2_S production. In order to verify whether the CSE-H_2_S pathway is the main pathway to produce the H_2_S, we used PAG as the irreversible inhibitor to CSE. Figure 3 showed that the intensity of fluorescence was decreased, which means the H_2_S was decreased, because of the pretreatment of the inhibitor, and the intensity of PAG group was as weak as the control group, indicating that the production of endogenous H_2_S was significantly inhibited with CSE inhibitor.

**Figure 4:**
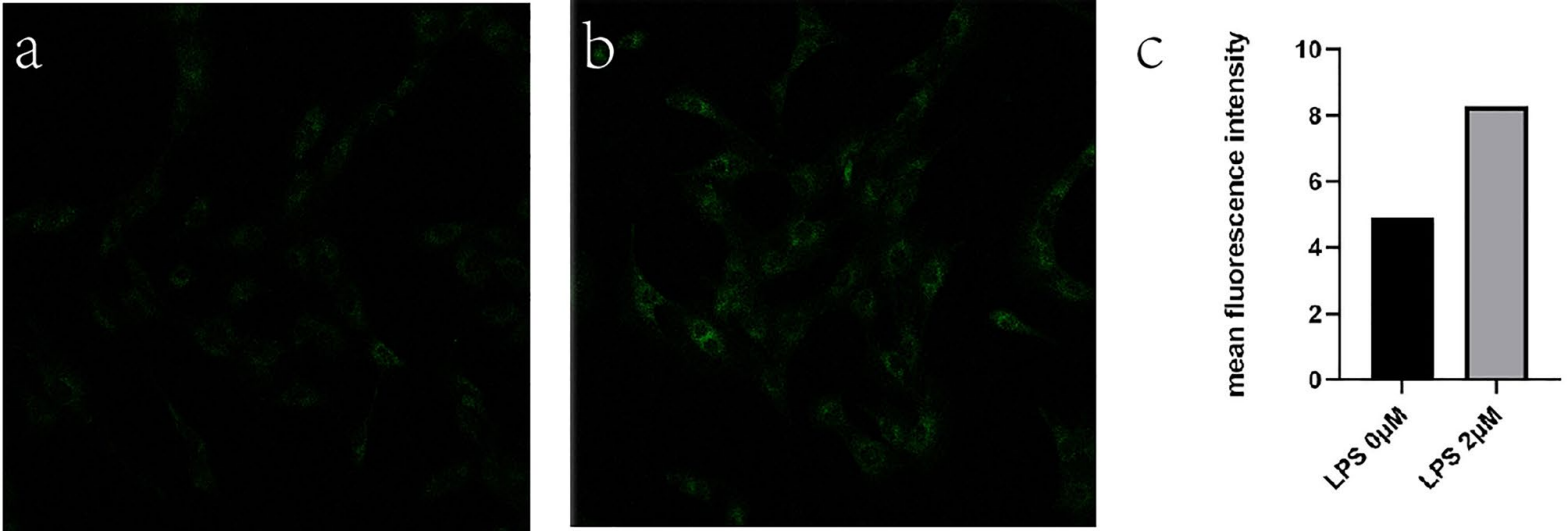
Cell fluorescence imaging of LPS induced endogenous H_2_S. (**a**) LPS 0 μM as control group. (**b**) Fluorescence image of MC3T3-E1 cells incubated with LPS (2 μM) for 30 min, then incubated with H_2_S probe (10 μM) for 30 min. (**c**) Fluorescence intensity analysis.

Cell fluorescence imaging of LPS induced endogenous H_2_S: when cells were treated with LPS (2 μg/mL) to produce inflammation, as shown by Fig. 4, intensive green fluorescence were produced compared to the control group. This indicates that when inflammation occurs, a lot of H_2_S was produced. In other words, the increase of H_2_S level can serve as an indicator for cells that are under the inflammation state. The production of endogenous H_2_S induced by lipopolysaccharide-mediated inflammation was successfully monitored with this H_2_S probe.

## Discussion

The main aim of the experiment is to solve the problem of detection of the inflammation of osteoblast, furthermore, we found that H_2_S produced by osteoblast is mainly via CSE-H_2_S pathway. In our study, we proved that our probe can be used in the normal cell to detect the H_2_S changes, which is rarely studied. There are already a lot of fluorescent probes that have been devised to detect intracellular H_2_S levels, however, to our knowledge, most of these probes were successfully applied to show alteration of H_2_S levels of tumor cells or living animals^15–17^. But in our study, we used a novel fluorescent probe to detect alteration of H_2_S levels in living osteoblast cells with exogenous or endogenous H_2_S for the first time. The H_2_S probe possesses high sensitivity, selectivity, and an ultrafast response to H_2_S, rendering it suitable for detection of H_2_S concentration in living cells. In order to determine whether the cell could translate Cys to H_2_S, and whether the probe could visualize endogenous H_2_S, we treated the osteoblast cells with irreversible inhibitor, PAG. The result proved that H_2_S is produced mainly by CSE-H_2_S pathway, which had not been proved in a visual way before. Other researchers have proved that (CSE) majorly contributed to endogenous H_2_S production in the primary osteoblast by overexpression and knockdown CSE^18^. This is consistent with our results.

For the inflammation of bone, there are two proved sources of H_2_S: bacteria and macrophage. When inflammation occurs, some bacteria produced and released H_2_S, including various common gram-negative pathogens in osteomyelitis such as Escherichia coli, Enterococcus faecalis, Enterobacter cloacae, and Klebsiella Pneumoniar. For macrophage, research shows that the level of H_2_S was improved and the expression of CSE mRNA increased because of the stimulate of LPS^19^. Our study shows that osteoblasts is the third source of H_2_S. Different sources of H_2_S might have interaction effect, for example, H_2_S production by osteoblast might modulate macrophage polarization and contribute to bone reparation. Keeping physiological level of endogenous H_2_S in PDLSCs/periodontal tissue is beneficial to maintain the homeostasis of periodontal tissue^20^. An appropriated level of H_2_S may play a vital role in maintaining the homeostasis of the bone marrow system. A previous study has clarified that BMSCs can produce H_2_S, regulate osteogenic differentiation and cell self-renewal, and that the lack of H_2_S could lead to defects in their differentiation^21^. Exogenous H_2_S could protect cell injury by regulating oxidative stress, mitochondrial function, and inflammation. While when inflammation occurs, H_2_S from bacteria disturbs the endogenous H_2_S of osteoblast cells, leads to a negative effect. In periodontitis studies, drugs that can release H_2_S have been used for the treatment, such as ATB-352, a kind of ketoprofen that can releasing H_2_S. The main aim is to minimize the presence of side-effect at the gastrointestinal tract. Meanwhile they found that the reduction of the inflammation even had a beneficial effect on bone resorption or tissue damage. ATB-346, releasing H_2_S like ATB-352, is beneficial for improving bone quality too^10^. Since H_2_S also can promote the development of periodontitis, there are still many questions about the biological mechanisms of H_2_S. It is well-know that there are many kinds of cell playing important roles in periodontitis, such as periodontal ligament stem cells, osteoclasts, and immune cells. Independent detection of H_2_S changes in living cell might facilitate the study of the role of H_2_S in diseases.

It was found previously that CBS and CSE were both increased in human gingival tissue during periodontitis through the technology of PCR and Western blot. However, H_2_S level or H_2_S synthesis in gingivitis and periodontitis was detected not increase after tissue homogenate^22^. This can be problematic for many reasons, such as the synthesis capacity decreased or consume increased of H_2_S in inflammation. But as a gasotransmitter, half of H_2_S can escape from medium in five minutes in tissue culture wells, which makes it hard to detect^23^. Under physiological conditions, H_2_S presents in three chemical ionization forms, about 18.5% H_2_S, 81.5% HS^−^ and minute quantities of S^2−^^24^. Different detection methods might lead to different results. H_2_S is more permeable in plasma membranes, the solubility of H_2_S in lipophilic solvents is quintuple greater than in water^25^, thus, fluorescence probe in theory could detect H_2_S more precisely. Our H_2_S probe might help deepen studies of the changes of H_2_S level and promote the progression on the researches about pathogenesis of periodontitis.

Fluorescence techniques is gaining widespread attention as sensors offering excellent sensitivity, good selectivity, and rapid response to changes. First of all, our probe has been shown to be sensitive for endogenous H_2_S detection and real-time monitoring of the changes in H_2_S in living cells, and it reacts quickly under physiological conditions. There are some things that can be improved, for example, a more precise target of probes to certain subcellular organelles, certain cells, tissues, or organs, which may be achieved by using near-infrared emit to get a greater tissue penetration and minimize the interference from background auto-fluorescence^26^. The probe might be improved, like detect Hcy/Cys/GSH/H_2_S at the same time^27^. For clinical use, H_2_S has a potential to be used as an appropriate biomarker for the related investigations of inflammation response. However, it still requires further development.

## Conclusion

In conclusion, it is the first experiment using H_2_S probe to detect H_2_S changes under stimulation in osteoblast in real time. We used a new hypotoxic H_2_S probe for exogenous and endogenous H_2_S detection in living osteoblast cells. Moreover, the results indicate that in osteoblast cells, H_2_S is produced mainly by CSE-H_2_S pathway directly, it also shows that under inflammation stimulation, endogenous H_2_S production will increase. The results suggest that H_2_S could be a potential marker for diagnosis of inflammatory diseases of bone, and it might help further studies for understanding the synthesis and change of H_2_S level in pathogenesis of periodontal disease.

## Data Availability

The datasets generated during the current study are available from the corresponding author on reasonable request.

## References

[1] Xu W, Zhou W, Wang H, Liang S (2020). Roles of porphyromonas gingivalis and its virulence factors in periodontitis. ADV Protein Chem. Str..

[2] Cheng R (2017). Porphyromonas gingivalis-derived lipopolysaccharide combines hypoxia to induce caspase-1 activation in periodontitis. Front. Cell Infect..

[3] Terrier CSP, Gasque P (2017). Bone responses in health and infectious diseases: A focus on osteoblasts. J. Infect..

[4] Thammasitboon K, Goldring SR, Boch J (2006). A role of macrophages in LPS-induced osteoblast and PDL cell apoptosis. Bone.

[5] Yoshinaga Y (2012). Topical application of lipopolysaccharide into gingival sulcus promotes periodontal destruction in rats immunized with lipopolysaccharide. J. Periodontal Res..

[6] Barbarra R (2011). Hydrogen sulfide generation in mammals: The molecular biology of cystathionine-Β- synthase (CBS) and cystathionine-Γ-lyase (CSE). Inflamm. Allergy Drug Targets..

[7] Yaegaki K (2008). Oral malodorous compound causes apoptosis and genomic DNA damage in human gingival fibroblasts. J. Periodontal Res..

[8] Wang H (2020). Hydrogen sulfide promotes lipopolysaccharide-induced apoptosis of osteoblasts by inhibiting the AKT/NF-κB signaling pathway. Biochem Biophys. Res. Co..

[9] Zs X (2011). Hydrogen sulfide protects MC3T3-E1 osteoblastic cells against H2O2-induced oxidative damage-implications for the treatment of osteoporosis. Free Radic. Biol. Med..

[10] Herrera BS (2015). The H2S-releasing naproxen derivative, ATB-346, inhibits alveolar bone loss and inflammation in rats with ligature-induced periodontitis. Med. Gas Res..

[11] Ibrahim H, Serag A, Farag MA (2021). Emerging analytical tools for the detection of the third gasotransmitter H2S, a comprehensive review. J. Adv. Res..

[12] Yang G (2008). H2S as a physiologic vasorelaxant: hypertension in mice with deletion of cystathionine gamma-lyase. Science.

[13] Yang B (2013). Evidence to challenge the universality of the horiuti-polanyi mechanism for hydrogenation in heterogeneous catalysis: Origin and trend of the preference of a non-horiuti-polanyi mechanism. J. Am. Chem. Soc..

[14] Ma Z (2020). Facile preparation of MnO2 quantum dots with enhanced fluorescence via microenvironment engineering with the assistance of some reductive biomolecules. ACS Appl. Mater. Interfaces..

[15] Wang J, Yu H, Li Q, Shao S (2015). A BODIPY-based turn-on fluorescent probe for the selective detection of hydrogen sulfide in solution and in cells. Talanta.

[16] Ji Y (2018). A novel BODIPY-based fluorescent probe for selective detection of hydrogen sulfide in living cells and tissues. Talanta.

[17] Xiong J (2018). Cyanine-based NIR fluorescent probe for monitoring H2S and imaging in living cells and in vivo. Talanta.

[18] Zheng Y (2017). Cystathionine γ-lyase-hydrogen sulfide induces runt-related transcription factor 2 sulfhydration, thereby increasing osteoblast activity to promote bone fracture healing. Antioxid. Redox. Sign..

[19] Badiei A, Rivers-Auty J, Ang AD, Bhatia M (2013). Inhibition of hydrogen sulfide production by gene silencing attenuates inflammatory activity of LPS-activated RAW264.7 cells. Appl. Microbiol. Biotechnol..

[20] Su Y (2015). Physiologic levels of endogenous hydrogen sulfide maintain the proliferation and differentiation capacity of periodontal ligament stem cells. J. Periodontol..

[21] Liu Y (2014). Hydrogen sulfide maintains mesenchymal stem cell function and bone homeostasis via regulation of Ca(2+) channel sulfhydration. Cell Stem Cell.

[22] Chun-Mei J (2017). Production of endogenous hydrogen sulfide in human gingival tissue. Arch. Oral. Biol..

[23] Cao X (2019). A review of hydrogen sulfide synthesis, metabolism, and measurement: Is modulation of hydrogen sulfide a novel therapeutic for cancer?. Antioxid. Redox. Signal..

[24] Hughes MN, Centelles MN, Moore KP (2009). Making and working with hydrogen sulfide: The chemistry and generation of hydrogen sulfide in vitro and its measurement in vivo: A review. Free Radic. Biol. Med..

[25] Jiang XW (2018). The expression of endogenous hydrogen sulfide signal during distraction osteogenesis in a rabbit model. Int. J. Oral. Maxillofac. Surg..

[26] Peng X (2005). Heptamethine cyanine dyes with a large stokes shift and strong fluorescence: A paradigm for excited-state intramolecular charge transfer. J. Am. Chem. Soc..

[27] Yin G (2021). Direct quantification and visualization of homocysteine, cysteine, and glutathione in Alzheimer's and Parkinson's disease model tissues. Anal. Chem..

